# (2*S*)-2-(3-Oxo-1,4-dioxaspiro­[4.5]decan-2-yl)ethanoic acid

**DOI:** 10.1107/S160053680801146X

**Published:** 2008-04-30

**Authors:** Yow-Fu Tsai, Yu-Ting Su, Chia-Her Lin

**Affiliations:** a Department of Chemistry, Chung-Yuan Christian University, Chung-Li 320, Taiwan

## Abstract

The title compound, C_10_H_14_O_5_, is an inter­mediate in our study of the asymmetric synthesis of α-hydroxy­alkanoic acids. The structure consists of 1,4-dioxaspiro[4,5]decane skeleton formed when the cyclohexylidene group binds to both of the hydroxyl groups of carboxylic groups of the starting malic acid. The six-membered ring adopts a chair conformation.

## Related literature

For related literature, see: Coppola & Schuster (1997 ▶); Díez *et al.* (2001 ▶); Dixon *et al.* (2005 ▶); Hanessian *et al.* (1993 ▶); Heimgartner & Obrecht (1990 ▶); Horgen *et al.* (2000 ▶); Liang *et al.* (2000 ▶); Sitachitta *et al.* (2000 ▶); Sugiyama *et al.* (1990 ▶).
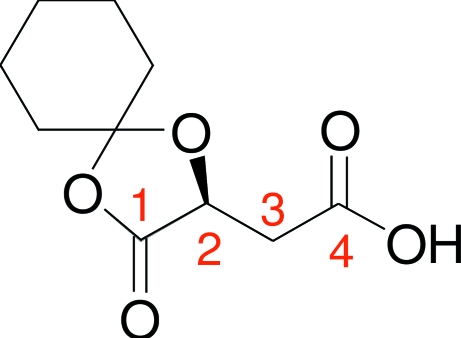


## Experimental

### 

#### Crystal data

C_10_H_14_O_5_
*M* *_r_* = 214.21
Orthorhombic, 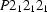

*a* = 6.7098 (6) Å
*b* = 10.3463 (8) Å
*c* = 15.3175 (13) Å
*V* = 1063.37 (15) Å^3^
*Z* = 4
Mo *K*α radiation
μ = 0.11 mm^−1^
*T* = 295 (2) K
0.50 × 0.45 × 0.35 mm

#### Data collection

Bruker Kappa APEXII CCD diffractometer
Absorption correction: multi-scan*SADABS*; Bruker, 2004 ▶) *T*_min_ = 0.948, *T*_max_ = 0.963
7861 measured reflections
2206 independent reflections
1814 reflections with *I* > 2σ(*I*)
*R*_int_ = 0.022

#### Refinement

*R*[*F* ^2^ > 2σ(*F* ^2^)] = 0.041
*wR*(*F* ^2^) = 0.123
*S* = 1.05
2206 reflections
138 parameters
H-atom parameters constrained
Δρ_max_ = 0.31 e Å^−3^
Δρ_min_ = −0.17 e Å^−3^

### 

Data collection: *APEX2* (Bruker, 2004 ▶); cell refinement: *APEX2*; data reduction: *SAINT* (Bruker, 2004 ▶); program(s) used to solve structure: *SHELXS97* (Sheldrick, 2008 ▶); program(s) used to refine structure: *SHELXL97* (Sheldrick, 2008 ▶); molecular graphics: *SHELXTL* (Sheldrick, 2008 ▶); software used to prepare material for publication: *SHELXTL*.

## Supplementary Material

Crystal structure: contains datablocks I, global. DOI: 10.1107/S160053680801146X/wk2081sup1.cif

Structure factors: contains datablocks I. DOI: 10.1107/S160053680801146X/wk2081Isup2.hkl

Additional supplementary materials:  crystallographic information; 3D view; checkCIF report

## Figures and Tables

**Fig. 1.**
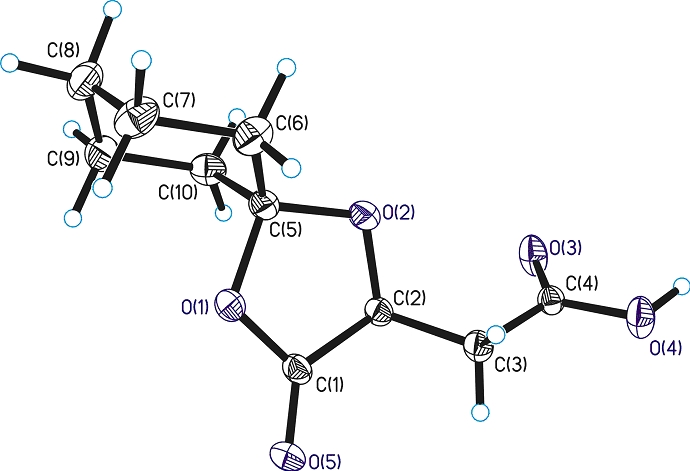
The molecular structure of the title compound, showing the atom numbering scheme. Displacement ellipsoids for non-H atoms are represented at the 30% probability level. The H atoms are drawn with an arbitrary radius.

## supplementary crystallographic information

### Comment

Enantiomerically pure α-hydroxy carboxylic acids are an important class of biological molecules (Liang *et al.*, 2000; Sitachitta *et al.*, 2000; Horgen *et al.*, 2000) as well as important intermediates for the synthesis of natural products (Coppola & Schuster, 1997; Sugiyama *et al.*, 1990; Heimgartner & Obrecht, 1990). For the above reasons, asymmetric synthesis of α-hydroxy carboxylic acids has attracted considerable attention. A number of synthetic strategies for preparing the optically active α-hydroxy carboxylic acids have been published in the literature (Dixon *et al.*, 2005; Díez *et al.*, 2001; Coppola & Schuster, 1997). The synthesis of the optically pure title compound ([*a*] ^20^_D_ = + 6.6°) (Scheme 1), which is an intermediate of our study on the asymmetric synthesis of α-hydroxyalkanoic acids, was carried out according to the reported method (Hanessian *et al.*, 1993) starting with the commercial optical pure *L*-(-)-malic acid. Herein, we report the single-crystal structure (Fig. 1) of the title compound. The crude product was recrystalized from ethyl acetate – *n*-hexane at room temperature, which allowed us to observe the single-crystal of the title compound. Notably, the cyclohexylidene group was bonded at the hydroxyl groups of carboxylic group (C-1) and on C-2 to show the spirocyclic structure.

### Experimental

Freshly distilled cyclohexanone (5.60 ml, 56.00 mmol) and BF_3_˙OEt_2_ (9.40 ml, 73.30 mmol) was added to a suspension solution of *L*-(-)-malic acid (5.01 g, 37.39 mmol) in dry ether (62.0 ml) cooled at 0 *^o^*C. The suspension gradually turned into a clear solution. After the mixture was stirred for 1 h at 0 *^o^*C, the ice bath was then removed and the mixture was stirred for 12 h at room temperature. The reaction mixture was diluted with ether and washed with 10% aqueous NaOAc (4 *x* 20.0 ml). The combined aqueous layers were extracted with ether, and the combined organic phases were washed three times with brine and dried over MgSO_4_. Removal of solvent in *vacuo* afforded a crude acid as pale yellow oil. Recrystallization (ethyl acetate /*n*-hexane) afforded 4.426 g (83%) of the acid 2 as an off-white crystal: *Rf* = 0.40 (ethyl acetate – *n*-hexane, 1/1, *v*/*v*); [*a*] ^21^_D_ = + 6.6° (c 1.2, CHCl3); ^1^H NMR (300 MHz, CDCl3) δ 9.29 (bs, 1H), 4.72 (dd, 1H, *J* = 6.3, 3.9 Hz), 2.99 and 2.86 (ABX, 2H, *J*_AB_ = 17.3, *J*_AX_ = 6.3, *J*_BX_ = 3.9 Hz), 1.89–1.30 (m, 10H).

### Refinement

The C-bound H atoms were placed in calculated positions (C-H = 0.97 - 0.98 Å) and included in the refinement in the riding-model approximation, with Uiso(H) = 1.2or 1.5U_eq_(C). The hydroxy H atoms were constrained to ideal geometries with O-H = 0.82 Å and Uiso(H) = 1.5U_eq_(O).

### Crystal data

**Table:**

C_10_H_14_O_5_	*F*_000_ = 456
*M**_r_* = 214.21	*D*_x_ = 1.338 Mg m^−^^3^
Orthorhombic, *P*2_1_2_1_2_1_	Mo *K*α radiation λ = 0.71073 Å
Hall symbol: P 2ac 2ab	Cell parameters from 3702 reflections
*a* = 6.7098 (6) Å	θ = 2.4–31.6º
*b* = 10.3463 (8) Å	µ = 0.11 mm^−^^1^
*c* = 15.3175 (13) Å	*T* = 295 (2) K
*V* = 1063.37 (15) Å^3^	Tabular, colourless
*Z* = 4	0.50 × 0.45 × 0.35 mm

### Data collection

**Table:**

Bruker Kappa APEXII CCD diffractometer	2206 independent reflections
Radiation source: fine-focus sealed tube	1814 reflections with *I* > 2σ(*I*)
Monochromator: graphite	*R*_int_ = 0.022
*T* = 295(2) K	θ_max_ = 33.3º
ω scans	θ_min_ = 2.4º
Absorption correction: multi-scan(SADABS; Bruker, 2004	*h* = −10→8
*T*_min_ = 0.948, *T*_max_ = 0.963	*k* = −15→9
7861 measured reflections	*l* = −13→22

### Refinement

**Table:**

Refinement on *F*^2^	Hydrogen site location: difference Fourier map
Least-squares matrix: full	H-atom parameters constrained
*R*[*F*^2^ > 2σ(*F*^2^)] = 0.041	*w* = 1/[σ^2^(*F*_o_^2^) + (0.0907*P*)^2^ + 0.0732*P*] where *P* = (*F*_o_^2^ + 2*F*_c_^2^)/3
*wR*(*F*^2^) = 0.123	(Δ/σ)_max_ < 0.001
*S* = 1.05	Δρ_max_ = 0.31 e Å^−^^3^
2206 reflections	Δρ_min_ = −0.17 e Å^−^^3^
138 parameters	Extinction correction: SHELXL97 (Sheldrick, 2008), Fc^*^=kFc[1+0.001xFc^2^λ^3^/sin(2θ)]^-1/4^
Primary atom site location: structure-invariant direct methods	Extinction coefficient: 0.077 (9)
Secondary atom site location: difference Fourier map	

### Special details

**Table:**

Geometry. All e.s.d.'s (except the e.s.d. in the dihedral angle between two l.s. planes) are estimated using the full covariance matrix. The cell e.s.d.'s are taken into account individually in the estimation of e.s.d.'s in distances, angles and torsion angles; correlations between e.s.d.'s in cell parameters are only used when they are defined by crystal symmetry. An approximate (isotropic) treatment of cell e.s.d.'s is used for estimating e.s.d.'s involving l.s. planes.
Refinement. Refinement of *F*^2^ against ALL reflections. The weighted *R*-factor *wR* and goodness of fit *S* are based on *F*^2^, conventional *R*-factors *R* are based on *F*, with *F* set to zero for negative *F*^2^. The threshold expression of *F*^2^ > σ(*F*^2^) is used only for calculating *R*-factors(gt) *etc*. and is not relevant to the choice of reflections for refinement. *R*-factors based on *F*^2^ are statistically about twice as large as those based on *F*, and *R*- factors based on ALL data will be even larger.

### Fractional atomic coordinates and isotropic or equivalent isotropic displacement parameters (Å^2^)

**Table:**

	*x*	*y*	*z*	*U*_iso_*/*U*_eq_	
O1	0.7046 (2)	0.66387 (12)	0.06930 (8)	0.0516 (3)	
O2	0.4717 (2)	0.51238 (14)	0.10628 (8)	0.0584 (4)	
O3	0.2465 (2)	0.28916 (12)	0.00641 (11)	0.0613 (4)	
O4	−0.0034 (2)	0.40271 (14)	−0.04693 (12)	0.0647 (4)	
H4A	−0.0620	0.3336	−0.0414	0.097*	
O5	0.6684 (3)	0.66532 (14)	−0.07558 (9)	0.0614 (4)	
C1	0.6310 (2)	0.61950 (15)	−0.00602 (11)	0.0438 (3)	
C2	0.4985 (3)	0.50524 (14)	0.01414 (10)	0.0406 (3)	
H2A	0.5662	0.4245	−0.0015	0.049*	
C3	0.2983 (3)	0.51228 (15)	−0.03022 (12)	0.0454 (3)	
H3A	0.2220	0.5827	−0.0051	0.055*	
H3B	0.3176	0.5309	−0.0917	0.055*	
C4	0.1827 (2)	0.38946 (15)	−0.02119 (10)	0.0393 (3)	
C5	0.6327 (3)	0.58484 (16)	0.14185 (11)	0.0461 (4)	
C6	0.5544 (4)	0.6724 (2)	0.21270 (13)	0.0615 (5)	
H6A	0.4875	0.6209	0.2568	0.074*	
H6B	0.4577	0.7317	0.1880	0.074*	
C7	0.7215 (5)	0.7482 (2)	0.25453 (14)	0.0702 (7)	
H7A	0.7760	0.8083	0.2122	0.084*	
H7B	0.6687	0.7981	0.3029	0.084*	
C8	0.8859 (4)	0.6613 (2)	0.28737 (14)	0.0686 (6)	
H8A	0.8347	0.6064	0.3336	0.082*	
H8B	0.9925	0.7136	0.3114	0.082*	
C9	0.9671 (3)	0.5778 (2)	0.21429 (14)	0.0643 (5)	
H9A	1.0289	0.6322	0.1704	0.077*	
H9B	1.0681	0.5200	0.2372	0.077*	
C10	0.8011 (3)	0.49950 (18)	0.17303 (13)	0.0547 (4)	
H10A	0.8540	0.4511	0.1240	0.066*	
H10B	0.7502	0.4381	0.2154	0.066*	

### Atomic displacement parameters (Å^2^)

**Table:**

	*U*^11^	*U*^22^	*U*^33^	*U*^12^	*U*^13^	*U*^23^
O1	0.0599 (8)	0.0417 (6)	0.0531 (6)	−0.0192 (6)	−0.0063 (6)	0.0093 (5)
O2	0.0631 (8)	0.0668 (8)	0.0454 (6)	−0.0280 (7)	−0.0004 (6)	0.0098 (6)
O3	0.0445 (6)	0.0356 (5)	0.1039 (11)	−0.0022 (5)	−0.0091 (7)	0.0121 (7)
O4	0.0425 (6)	0.0513 (7)	0.1003 (11)	−0.0049 (6)	−0.0158 (7)	0.0192 (7)
O5	0.0622 (8)	0.0654 (9)	0.0565 (7)	−0.0148 (7)	0.0016 (6)	0.0219 (7)
C1	0.0436 (7)	0.0370 (6)	0.0509 (8)	−0.0079 (6)	0.0021 (7)	0.0086 (6)
C2	0.0434 (7)	0.0330 (6)	0.0455 (7)	−0.0058 (6)	−0.0007 (6)	0.0048 (6)
C3	0.0436 (7)	0.0360 (7)	0.0568 (8)	−0.0043 (6)	−0.0036 (7)	0.0096 (6)
C4	0.0376 (6)	0.0365 (6)	0.0440 (7)	−0.0006 (6)	−0.0002 (6)	0.0009 (5)
C5	0.0516 (9)	0.0420 (7)	0.0448 (7)	−0.0098 (7)	−0.0003 (7)	0.0040 (6)
C6	0.0602 (11)	0.0677 (12)	0.0564 (10)	0.0122 (10)	−0.0036 (9)	−0.0061 (9)
C7	0.0984 (19)	0.0535 (11)	0.0588 (11)	0.0035 (11)	−0.0118 (12)	−0.0117 (9)
C8	0.0772 (15)	0.0712 (13)	0.0575 (10)	−0.0108 (12)	−0.0193 (10)	−0.0002 (10)
C9	0.0513 (10)	0.0734 (13)	0.0683 (11)	0.0032 (10)	−0.0065 (9)	0.0130 (11)
C10	0.0663 (11)	0.0425 (8)	0.0552 (8)	0.0047 (9)	0.0025 (9)	0.0053 (7)

### Geometric parameters (Å, °)

**Table:**

O1—C1	1.336 (2)	C5—C10	1.511 (3)
O1—C5	1.4617 (19)	C6—C7	1.511 (3)
O2—C5	1.423 (2)	C6—H6A	0.9700
O2—C2	1.425 (2)	C6—H6B	0.9700
O3—C4	1.199 (2)	C7—C8	1.510 (4)
O4—C4	1.317 (2)	C7—H7A	0.9700
O4—H4A	0.8200	C7—H7B	0.9700
O5—C1	1.193 (2)	C8—C9	1.515 (3)
C1—C2	1.511 (2)	C8—H8A	0.9700
C2—C3	1.507 (2)	C8—H8B	0.9700
C2—H2A	0.9800	C9—C10	1.515 (3)
C3—C4	1.495 (2)	C9—H9A	0.9700
C3—H3A	0.9700	C9—H9B	0.9700
C3—H3B	0.9700	C10—H10A	0.9700
C5—C6	1.508 (3)	C10—H10B	0.9700
			
C1—O1—C5	110.00 (12)	C7—C6—H6A	109.4
C5—O2—C2	108.11 (13)	C5—C6—H6B	109.4
C4—O4—H4A	109.5	C7—C6—H6B	109.4
O5—C1—O1	123.82 (15)	H6A—C6—H6B	108.0
O5—C1—C2	128.12 (16)	C8—C7—C6	111.94 (17)
O1—C1—C2	108.05 (13)	C8—C7—H7A	109.2
O2—C2—C3	109.37 (15)	C6—C7—H7A	109.2
O2—C2—C1	103.66 (13)	C8—C7—H7B	109.2
C3—C2—C1	113.23 (12)	C6—C7—H7B	109.2
O2—C2—H2A	110.1	H7A—C7—H7B	107.9
C3—C2—H2A	110.1	C7—C8—C9	110.88 (17)
C1—C2—H2A	110.1	C7—C8—H8A	109.5
C4—C3—C2	112.30 (13)	C9—C8—H8A	109.5
C4—C3—H3A	109.1	C7—C8—H8B	109.5
C2—C3—H3A	109.1	C9—C8—H8B	109.5
C4—C3—H3B	109.1	H8A—C8—H8B	108.1
C2—C3—H3B	109.1	C10—C9—C8	110.37 (19)
H3A—C3—H3B	107.9	C10—C9—H9A	109.6
O3—C4—O4	122.26 (15)	C8—C9—H9A	109.6
O3—C4—C3	125.67 (15)	C10—C9—H9B	109.6
O4—C4—C3	112.07 (14)	C8—C9—H9B	109.6
O2—C5—O1	104.72 (12)	H9A—C9—H9B	108.1
O2—C5—C6	109.10 (17)	C5—C10—C9	111.66 (15)
O1—C5—C6	109.04 (14)	C5—C10—H10A	109.3
O2—C5—C10	112.38 (15)	C9—C10—H10A	109.3
O1—C5—C10	108.68 (15)	C5—C10—H10B	109.3
C6—C5—C10	112.58 (15)	C9—C10—H10B	109.3
C5—C6—C7	111.0 (2)	H10A—C10—H10B	107.9
C5—C6—H6A	109.4		
